# Beliefs, knowledge, actions of nursing techniques in breastfeeding in pain management in immunization

**DOI:** 10.1590/0034-7167-2021-0546

**Published:** 2022-07-18

**Authors:** Isadora Trinquinato Rosa, Lisabelle Mariano Rossato, Danila Maria Batista Guedes, Vanessa Dias Fogaça, Flávia Domingues, Lucía Silva

**Affiliations:** I Universidade de São Paulo. São Paulo, São Paulo, Brazil; II Universidade Federal de São Paulo. São Paulo, São Paulo, Brazil

**Keywords:** Pain Management, Pediatric Nursing, Neonatal Nursing, Breast Feeding, Immunization., Manejo del Dolor, Enfermería Pediátrica, Enfermería Neonatal, Lactancia Materna, Inmunización., Manejo da Dor, Enfermagem Pediátrica, Enfermagem Neonatal, Amamentação, Imunização.

## Abstract

**Objective:**

Understand the beliefs, knowledge, and actions of nursing technicians on breastfeeding as a form of non-pharmacological intervention to relieve pain in newborns and infants during immunization.

**Methods:**

Qualitative study carried out through semi-structured interviews with nine nursing technicians from three Basic Health Units in a city in the state of São Paulo. The theoretical approach of the Belief Model and the methodological framework of Thematic Analysis supported this study.

**Results:**

Three themes originated: Beliefs, Knowledge, and Actions of nursing technicians.

**Final considerations:**

Despite knowledge about the benefits of breastfeeding as the most effective method for relieving pain in newborns and infants during vaccination, their restrictive beliefs overrode the evidence, leading them to act in ways that discourage or prevent the mother from breastfeed during vaccination. Formal training is recommended to align with current evidence-based practices.

## RESUMO

**Objetivo:**

Compreender as crenças, o conhecimento e as ações das técnicas de enfermagem sobre a amamentação como forma de intervenção não farmacológica no alívio da dor em recém-nascidos e em lactentes durante a imunização.

**Métodos:**

Estudo qualitativo realizado mediante entrevistas semiestruturadas com nove técnicas de enfermagem de três Unidades Básicas de Saúde de uma cidade do estado de São Paulo. A abordagem teórica do Modelo de Crenças e o referencial metodológico da Análise Temática ampararam este estudo.

**Resultados:**

Originaram-se três temas: Crenças, Conhecimento e Ações das técnicas de enfermagem.

**Considerações finais:**

Apesar do conhecimento sobre os benefícios da amamentação como o método mais eficaz para o alívio da dor em recém nascidos e lactentes durante a vacinação, suas crenças restritivas sobrepuseram-se à evidência, levando-as a agir de modo a desencorajar ou impedir a mãe de amamentar durante a vacinação. Recomenda-se treinamento formal para alinhamento de práticas atuais baseadas em evidências.

## RESUMEN

**Objetivo:**

Comprender creencias, conocimiento y acciones de técnicas de enfermería sobre la lactancia materna como forma de intervención no farmacológica en el alivio del dolor en neonatos y en lactantes durante la inmunización.

**Métodos:**

Estudio cualitativo realizado mediante entrevistas semiestructuradas con nueve técnicas de enfermería de tres Unidades Básicas de Salud de una ciudad de São Paulo. El abordaje teórico del Modelo de Creencias y el referencial metodológico del Análisis Temático ampararon este estudio.

**Resultados:**

Originado tres temas: Creencias, Conocimiento y Acciones de las técnicas de enfermería.

**Consideraciones finales:**

Aunque del conocimiento sobre los beneficios de la lactancia materna como el método más eficaz para el alivio del dolor en neonatos y lactantes durante la vacunación, sus creencias restrictivas antepuestas a la evidencia, llevándolas a actuar de modo a desanimar o impedir la madre de amamantar durante la vacunación. Recomendado entrenamiento formal para alineamiento de prácticas actuales basadas en evidencias.

## INTRODUCTION

Pain is a subjective and individual experience. It can be defined as “an aversive sensory and emotional experience typically caused by, or similar to that caused by actual or potential tissue damage”. Six main and etymological notes on pain are presented below: 1) pain is always a personal experience that is influenced to varying degrees by biological, psychological and social factors; 2) pain and nociception are different phenomena; 3) pain cannot be inferred only through the activity of sensory neurons; 4) through their life experiences, individuals learn the concept of pain; 5) the report of the experience of a person with pain must be respected; 6) although pain generally plays an adaptive role, it can have adverse effects on social and psychological function and well-being. Verbal description is just one of several behaviors for expressing pain; the inability to communicate does not negate the possibility that a human or non-human animal may experience pain^(1)^.

International studies show that newborns and infants suffer, during childhood, about 20 needlesticks, from 2 to 18 months of age, as a result of vaccines applied at this stage^(2-3)^. One way to understand the intensity and quality of pain is through: assessment scales, which make it possible to know and assess pain; and changes in vital signs and behavioral attitudes, such as crying, speech, and facial expressions, to determine their degree of discomfort and pain, as well as the degree of relief provided by medications or other non-pharmacological measures^(3-4)^.

Incorrect pain management or lack of management can have psychological, physiological, motor, cognitive and sensory consequences, and repeated exposure to pain can cause, in the long term, greater sensitivity and increased pain response^(2-3,5-6)^. Literature shows that newborns and infants remember pain. These memories create memories and influence their perceptions, their behaviors, and their needs during the subsequent experiences of pain to which they will be submitted, as in the case of immunization - which has its period from birth to adolescence, concentrating the greatest number of painful procedures. from 0 to 10 years of age^(3,7-8)^. Current evidence highlights that newborns and infants continue to experience pain unnecessarily, even when relief options exist, as a reflection of inadequate pain management practices by healthcare professionals^(7,9-10)^.

There is scientific evidence recommending breastfeeding during invasive procedures in full-term babies, considered the safest and most effective way to relieve pain compared to other non-pharmacological methods, such as: holding the baby in the lap; skin-to-skin contact; topical anesthetics; sweetened solutions; non-nutritive sucking; expressed breast milk; and music therapy^(2,8,11)^. Studies carried out in developed and developing countries show that, in the last decade, the prevention and management of pain in newborns is considered relatively adequate, but newborns and infants have not received adequate pain relief; because of this, studies recommend that more non-pharmacological methods of pain relief in newborns during invasive procedures be used in clinical practice^(3,6,12-15)^. A very common and accessible area for the implementation of non-pharmacological methods for pain relief in newborns and infants is immunization, performed in Basic Health Units (BHU) by nursing technicians, mostly.

In October 2021, the Technical Note of the Ministry of Health in Brazil was published on good breastfeeding practices as a non-pharmacological measure to reduce pain during the administration of injectable vaccines in children. It was recommended that health services favor and support the lactating mother in breastfeeding the child immediately before and during the administration of injectable and oral vaccines, so as not to prevent the mother from breastfeeding her baby during vaccination, even when oral vaccines are present^(16)^. Recent Brazilian studies corroborate the recent recommendation^(16)^. A survey on the perception of mothers about breastfeeding as a non-pharmacological method of pain relief shows that breastfeeding is still little used in practice during the time of vaccination and that, if performed, it would promote both the mother and the infant and newborn. A more pleasant environment was born, favoring the relief of anguish and pain^(17)^. Still in 2021, another national study published on this topic reinforces that breastfeeding during vaccine application is an efficient, simple, free, and easily accessible strategy for the management of pain in newborns and infants at this time^(18)^.

Therefore, it is necessary to carry out this study with the following research question: What are the beliefs of nursing technicians from Basic Health Units about the use of breastfeeding during immunization as a non-pharmacological intervention to reduce pain in newborns and infants?

## OBJECTIVE

Understand the beliefs, knowledge, and actions of nursing technicians on breastfeeding as a form of non-pharmacological intervention to relieve pain in newborns and infants during immunization.

## METHODS

### Ethical aspects

Data collection began after the project was approved by the Research Ethics Committee of the USP School of Nursing, with which the Basic Health Units agreed; and was carried out between August 2020 and January 2021. The study was based on Resolution 466, of December 12, 2012, of the National Health Council, Ministry of Health, which regulates research involving human beings. The Free and Informed Consent Term (FICT) was delivered and explained to the study participants by the researcher. To maintain anonymity and privacy, their names were replaced by alphanumeric codes, formed by the letter “E” (from “interviewee”), followed by a cardinal numeral (E1, E2…).

### Theoretical-methodological framework

The Illness Beliefs Model (IBM)^(19)^ was used as the theoretical approach to support the discussion on how the beliefs of nursing technicians facilitate or hinder the use of breastfeeding as a form of non-pharmacological intervention to reduce pain of newborns and infants during the vaccine procedure. This approach discusses the understanding of facilitating and restrictive beliefs in health professionals and family members^(19)^. The authors define “disease” as any physical, emotional, relational and/or spiritual suffering. It is worth mentioning that the world view of professionals is essential in the care provided, as it can open or close opportunities to reduce suffering in family members as a whole^(19)^. In this study, we consider nursing technicians as health or nursing professionals. Thematic Data Analysis^(20)^ was used as a methodological framework.

### Study type

This is an exploratory and descriptive study with a qualitative approach, which made use of semi-structured interviews with nursing technicians, following the recommendations of the Consolidated criteria for reporting qualitative research (COREQ)^(21)^.

### Study scenario

The research was carried out in three FHUs in the city of Jundiaí, in the interior of São Paulo, which has approximately 420 thousand inhabitants^(22)^. The city has 35 BHUs, with an average of four nursing technicians working in each one^(23)^. The choice of units was carried out by the city hall at random, and the research was authorized to the authors.

### Data sources

All professionals from the nursing team working in the BHUs (nurses, technicians, and nursing assistants) were invited to participate in the study, but only the nursing technicians agreed to participate in the study, these being those who worked directly in the vaccination rooms. The age of the nursing technicians ranged between 32 and 60 years. The time working in the area ranged from 4 to 28 years. Nursing technicians who worked in BHU directly in the application of vaccines in newborns and infants were included; and excluded those who were on maternity, health, or vacation leave.

### Data collection and organization

Initially, the researcher contacted the nursing coordinators of each BHU co-participating in this study by telephone and by e-mail, to present the research project and request authorization for data collection. The selection of nursing techniques was performed after approval by the coordination of each FHU. The interviews were scheduled with each nursing technician according to their availability and were carried out by the researcher in a private room, made available by the head of each unit, in order to guarantee the privacy of the interviewees. The interviews, which lasted an average of 30 minutes, followed the script developed for the study, consisting of identification, training, performance and professional qualification data, in addition to the guiding questions elaborated by the authors, which were: “What do you know? and believe about breastfeeding in newborns and infants during immunization?” and “Do you perform any practice as a non-pharmacological intervention to reduce pain at the time of immunization?” Data collection was terminated after theoretical data saturation.

### Data analysis

The data were collected and transcribed simultaneously, with transcriptions and records of the speeches of each study participant being carried out. The researcher followed the six steps for the thematic analysis, proposed by Braun & Clarke^(20)^: 1) Getting familiar with their data - The interviewees’ responses were recorded by the researcher, using a cell phone voice recorder, and transcribed in full to aid in continuous reading and re-reading; 2) Generating initial codes - The coding of data characteristics was carried out systematically, across the entire data set; 3) Searching for themes - The coding of data allowed the definition of themes and sub-themes, which were analyzed for the production of the final report; 4) Reviewing themes - It was verified that the coding was in accordance with the themes, generating thematic map; 5) Defining and naming themes - Refinement of the specifics of each theme through new analysis; 6) Producing the report - Relationship between analysis, research question and literature, producing an academic report of the analysis^(24-25)^.

## RESULTS

The results obtained in the analysis of the interviews were organized into three themes and seven subthemes. To maintain the anonymity and privacy of the participants, their names were replaced by alphanumeric codes, formed by the letter “E” (from “interviewee”), followed by a cardinal numeral (E1, E2…).

Theme 1 (Facilitating and Restricting Beliefs of Nursing Techniques), subdivided into two subthemes, identified the statements that encompass: the beliefs of nursing technicians in relation to the newborn and infant, and their pain; and the personal beliefs of the nursing technicians about other aspects that could or could not influence breastfeeding during the application of the vaccine as a method of pain relief.

### Facilitating beliefs of nursing techniques that influence breastfeeding during vaccination

The beliefs of nursing technicians about breastfeeding are relevant to understand breastfeeding as something positive, and this can be applied to newborns and infants:

*I have my own experience: my mother breastfed me until I was 3 years old. I am grateful to my mother, who for the rest of eternity, she had the affection, she had, you know, that availability... she was a person who worked, but who did everything to be able to breastfeed. A person who, at the time, had no information, no media, no hospital, nothing. More than 50 years ago! So, I see my mother, now deceased, the respect I have, the respect for this love, for this affection that she could give me, it was different for me.* (E2)

Regarding the reception of the newborn and infant, some interviews showed that the nursing technicians believed that welcoming and talking directly to the newborn and infant could be a facilitating strategy for pain relief during vaccination:

*But it’s this conversation, it’s the reception, it’s explaining the effect of each vaccine, which we do very carefully, talking to the child - even the little ones. When we did the BCG here,* […] *we talked to the child, we talked to the baby, and so we play, we talk, making that exchange! So, always leaving the child in evidence, her feeling, respecting.* (E1)

During the interviews, some nursing techniques demonstrated facilitating beliefs such as effective non-pharmacological pain relief interventions. They observed that both the reception and the permission of breastfeeding at the time of administering the vaccine could have a positive influence, promoting a welcoming, safe, and comfortable environment:

*I think that the baby does not associate the pain of the sting with being on the breast, so at the next feeding he does not want to breastfeed, because he thinks he will suffer again. I don’t think he has that association. That’s why we leave it, because poor people, it’s so instantaneous for you to bite and let go of the sippy cup, that I think he won’t even remember that he was in the sippy cup at the time he stung.* (E2)

### Restrictive beliefs of nursing techniques in the face of newborn and infant pain, which influence breastfeeding during vaccination

During the interviews, it was possible to identify that each technique considered the pain level of the newborn and infant based on the behavior and intensity of crying at the time of vaccination:

*I think it all comes together. When the baby feels a lot of pain, you notice that, in addition to crying, that strong, high-pitched cry, he closes his hands, and you know he is hurting. Every baby reacts in a different way, but most are the high-pitched cry-that loudest cry-and the little clenched hand. Because of the baby’s reaction to the vaccine, we can assess which vaccine hurts more, which hurts less, precisely because of his reaction.* (E6)

Some points of view could have a negative influence, such as restrictive beliefs in the absence of breastfeeding during the application of the vaccine: the speeches of certain nursing technicians showed that they were against breastfeeding as a method of pain relief:

*My privacy? No* [does not think the mother should breastfeed during vaccination]. *Not because - I have a daughter who is pregnant - and I already have two grandchildren. I already told her: “Do not breastfeed when giving the vaccine”. Because I think that, when the baby breastfeeds, it must be a pleasant time, it has to be something pleasant for him, calm, and he can relate breastfeeding with pain! He understood?! And this I have with me. That’s what I advised my daughter: “No… give the vaccine later; if you want to please, if you want to breastfeed, then you do it.”* [...] *I believe I associate pain with breastfeeding.* (E8)

In addition to these statements, the fear of the newborn and infant choking during the vaccination was one of the restrictive beliefs presented during the interviews.:

*In my opinion, well, privately, I think it’s even - I’m not going to say it’s going to happen - but that it’s a risk. Because, if he has milk in his throat and starts crying, I think he’s even a little scared to choke!* (E1)

Aside from the feeling of fear and the belief that the newborn and infant could choke during the application of the vaccine, many nursing technicians clearly demonstrated their restrictive belief that breastfeeding as a method of pain relief it is ineffective or negative, because they believe that if he lets go of the mother’s breast during the procedure, it does not relieve his pain:

*I think not. First, I didn’t notice any difference. The baby cries, he cries when breastfeeding, sometimes he lets go of the breast, lets go, comes back, but he continues to cry with the breast in his mouth.* (E8)

*But we are not successful with that, no. Because what we have noticed: you put the baby to breastfeed when you are going to have the injectable; the needle arrived, it doesn’t suck at all! They feel anyway, even breastfeeding. There’s no way, currently it doesn’t calm down.* (E2)

Specifically in relation to the rotavirus vaccine, many nursing technicians, who had the restrictive belief that the newborn and infant could regurgitate during the procedure, negatively influenced the mother not to breastfeed during vaccine application.:

*Rotavirus is two and four months. What happens: in the literature, there are no contraindications. However, the child, by the effort of crying, can regurgitate. It’s more this question of the child ending up regurgitating. If the mother can wait a little bit… It’s ideal.* (E5)

In Theme 2 (Knowledge of nursing techniques), the participants demonstrated knowledge about general methods of relieving pain in newborns and infants during the application of vaccines; among these, breastfeeding or other possible methods. In this theme, two subthemes were identified, described below.

### Knowledge of nursing techniques on breastfeeding as a non-pharmacological intervention for pain relief

During the interviews, only three of the nine study participants demonstrated knowledge about breastfeeding as a non-pharmacological method of pain relief at the time of administering the vaccine. Some had doubts about the possibility of the mother being able to breastfeed during vaccination, due to the lack of formal guidelines on the safety of breastfeeding during this procedure:

*There is even a manual from the Ministry of Health that says that if the mother wants to breastfeed to calm the child, no problem. There are some who want. Maybe it’s a time for me to be more focused and to be talking to her about the vaccines, about the return, to say: “You can put it on the chest so he can calm down.” Do not. I never had that orientation either. So far, it hasn’t been said like this: “It’s to guide breastfeeding at the moment” or “It’s not to leave, because there is a risk of something.” We were never advised not to breastfeed, so…* (E3)

### Knowledge of nursing techniques about other pain relief methods

The nursing technicians mentioned certain devices brought by the mothers during the application of the vaccine or in relation to other forms and techniques of pain relief and reduction of side effects:

*There are mothers who come here with little devices that have little teeth and ask us to use them, a little plastic thing, with little teeth. He says that the child does not feel much pain from the vaccine. I don’t know if it’s the mother’s psychological or if it’s scientifically proven; I haven’t read about it yet, but there are some mothers who come with this little device, to be able to relieve the moment of pain.* (E3)

In Theme 3 (Actions of nursing technicians), reports on the actions of nursing technicians regarding the pain of newborns and infants during vaccine application are included. This theme was divided into three sub-themes, described below.

### Facilitating and restrictive actions of nursing techniques in relation to pain relief for newborns and infants

Few nursing technicians have commented on their direct action to relieve pain in newborns and infants during vaccination. Some believed that they could not provide pain relief, due to the lack of resources in the unit:

*Look… I, here, don’t use anything to ease the pain. At the post, we don’t have it. It doesn’t have that custom either.* (E8)

As for the intervention aimed at pain relief, some nursing technicians commented during the interviews about the pharmacological guidance for pain relief:

*We also advise, in terms of getting very sore, giving a few drops that the pediatrician has already given, to have this care at home* [antipyretic]. (E2)

Welcoming the newborn and infant before the vaccine application represented the care actions of the nursing technicians with them, explaining how the procedure would be performed, even if they did not understand exactly the meaning of each word said; and distracting them:

*They already start to cry, and I always try to play a little game, distract them a little, even wait a little while to vaccinate.* (E9)

After the application of the vaccine, the action of welcoming the child through the guidance of the mother to breastfeed after the vaccine was adopted by the nursing technicians as a non-pharmacological form of pain relief.:

*So, we advise the mother for after finishing: “Mom, sit outside for a little while, feed the baby, then he will calm down.”* (E2)

### Facilitating actions of nursing techniques in relation to the safe technique of vaccine application

Vaccine safety, through the correct positioning of the newborn and infant on the parents’ lap, emerged during the interviews as an important factor, because, when correctly positioned, it was easier for the nursing technician to apply the vaccine. The snuggle was kept on the mother’s lap, which is more comfortable and presents less risk:

*The family member holds it, we arrange it on the lap, teaches how to hold it so as not to hurt the baby. Because the little ones don’t, but, after a certain age, they get stronger, they pull, when they’re older they do it with their hand to pull the syringe out of their hand, so we teach the family member to hold it, for protection of child.* (E4)

### Restrictive actions of nursing techniques when the mother wants to breastfeed

This subtheme highlights the technique’s response when the mother verbalizes the desire to breastfeed during vaccination, whether orally, intramuscularly, or subcutaneously. Nursing technicians showed some divergences in guidelines regarding whether or not to breastfeed during, before or after the administration of the rotavirus dose:

*I cannot repeat the dose of rotavirus, so it may happen that I miss that dose as well. If I happen to be breastfeeding, or even come with a full stomach, I get the vaccine and the child regurgitates, I cannot reapply the vaccine!* (E5)

*The more he has an empty stomach, the better, because there is a risk that the child will regurgitate. So we let you know. But sometimes they already enter the vaccine room with the infant suckling. There are no contraindications, but what we warn you is: if the child regurgitates or vomits, we no longer apply it at that time.* (E3)

When the mother says that she wants to breastfeed during the injection of the vaccine, the response can vary between nursing technicians who are not bothered by the mother breastfeeding during vaccination and those who are opposed to the practice:

*I always tell her: “You are at ease; if you want to put him on the breast, you can let him breastfeed; get ready in case he chokes or something, so you can get him off your chest.” So I tell her: “Take it off the chest while I apply the vaccine, then you put it on.” I always leave it to the mother’s discretion. For me, it is indifferent, it never got in the way of the procedure.* (E9)

*If she asks me “What do you think?” (About breastfeeding during the administration of the injectable vaccine), then I say: “I think it’s bad.” Breastfeeding has to be pleasurable; the moment is peaceful. Then he is breastfeeding and in pain. Like it or not, he’s going to feel it, poor thing.* (E8)

## DISCUSSION

The narratives presented in Theme 1 refer to the beliefs of nursing technicians, which can be both facilitative and restrictive regarding how these beliefs can interfere with pain relief for newborns and infants - with emphasis on the fact that there is this free and non-pharmacological option during the time of vaccination.

Some nursing techniques, during Theme 1, presented positive beliefs about breastfeeding as a non-pharmacological method of pain relief and used welcoming attitudes. This action is in accordance with the recommendation of the World Health Organization (WHO)^(26)^ that welcoming newborns and infants and the family can help control pain, which validates the beliefs of the nursing technicians in this study. The WHO^(26)^ also presents some measures of professional guidance on pain reduction during the application of the vaccine, such as ensuring the presence of the caregiver with the baby at the time of the vaccine; allow children to be held on their laps during the procedure; promote breastfeeding before vaccination and/or during the procedure^(26)^.

Still in Theme 1, some nursing technicians had restrictive beliefs and were against breastfeeding as a non-pharmacological intervention for pain relief, as they do not understand that pain relief does not mean the absence of crying; therefore, they could not identify the relief. In fact, even with the newborn and infant sucking, when they receive a painful sting, they may pause, cry opening their mouth and return to the chest to calm down, representing pain relief. It so happens that the reduction in crying time, heart rate and the decrease in the interval to return to suckling after the bite are effective strategies to assess pain reduction, so that, based on evidence of this type, several recent studies prove that breastfeeding is the most effective non-pharmacological intervention for pain relief^(2,15,27-29)^.

In Theme 1, the subtheme “Restrictive beliefs of nursing technicians in the face of newborn and infant pain, which influence breastfeeding during vaccination” presents the fear of choking and other complications as restrictive beliefs of nursing techniques, which can have a negative impact, preventing the pain relief of the newborn and infant during the application of the vaccine. Results of systematic reviews show that, despite the fear of health professionals or nurses regarding choking, no complications related to breastfeeding were reported during invasive procedures, suggesting, therefore, that there is no risk of airway compromise, such as coughing, choking and aspiration^(15,28)^.

Regarding the possibility of the baby regurgitating the rotavirus vaccine, according to the guidelines contained in the package insert of the vaccine most used by the SUS, it is not necessary to wait any period between breastfeeding and administration of the vaccine^(30)^.

According to studies, the lack of professional knowledge about breastfeeding as a non-pharmacological strategy for pain relief in invasive procedures can be a restrictive belief and an obstacle for the mother to breastfeed, depriving the newborn and infant of pain relief^(27,31)^.

The authors of the Belief Model^(19)^ argue that some beliefs can be considered more useful than others: useful beliefs are called “facilitating beliefs”; and less useful beliefs, called “restrictive beliefs”. They mention that there are no “good or bad” beliefs, but useful beliefs in professional-patient relationships and in coping strategies for certain situations. There may be a situation where the belief is facilitating for one person (e.g., the technique that does not oppose or rely on scientific evidence and allows the mother to breastfeed her baby) but may be restrictive for another person (e.g. .ex., the technique that refuses to guide breastfeeding during vaccination, for not believing in science, depriving the mother of relieving her child’s pain). Restrictive beliefs reduce the possibility for the health professional to discover or solve challenges and problems - such as, in this case, the pain of the newborn and infant at the time of vaccine application. The fact that the nursing technique is against breastfeeding as a non-pharmacological intervention for pain relief increases the suffering of newborns and infants, by depriving them of free, fast, safe, and available pain relief through breastfeeding. Facilitating beliefs, on the other hand, increase the possibility for the nursing technician to seek solutions for pain relief - such as allowing breastfeeding during vaccination.

The adequate management of pain is considered, by the National Council for the Defense of the Rights of Children and Adolescents, as a fundamental human right. In Brazil, newborns and infants have the right not to feel pain when there are means to avoid it, guaranteed by law^(32)^.

In Theme 2 (Knowledge of nursing techniques), some of them recognized breastfeeding as a possibility of pain relief; while others were unaware of this possibility, claimed that this practice was not recommended and were confused about whether or not to guide breastfeeding during the procedure, due to the lack of formal guidance on the subject.

It is important to note that the role that nursing technicians’ beliefs play in the process of using knowledge supports the use of breastfeeding and serves as a basis for promoting changes in nursing practice^(15)^. The Child’s Handbook^(33)^ - a document offered free of charge by the SUS in the form of a booklet, distributed in maternity hospitals or BHUs - contains information and specific spaces for nursing technicians to fill in the child’s vaccination record, being an up-to-date informational tool of the health. In this document, the guidance is clearly described for the mother to breastfeed her child during the application of any type of vaccine. This booklet is taken by the parents to the health service every time the child is vaccinated; therefore, both parents and nursing technicians can have access to this information before vaccination in all vaccine application opportunities^(33)^.

As for knowledge about other non-pharmacological interventions for pain relief, some nursing technicians mentioned devices brought by the mother, who believed to provide relief to the newborn and infant, and the nursing technician had doubts about the effectiveness of these items. Therefore, although nursing technicians are aware of the baby’s pain, they are often unable to implement strategies in a systematic way in order to improve pain management^(34)^.

When the infant is subjected to pain, he usually has an increase in heart and respiratory rate, sweating in the palms of the hands and soles of the feet (occurs by the activation of the sympathetic nervous system in response to emotional stimuli such as pain and anxiety), facial expressions of pain (squinting, crying, frowning, clasping hands), high-pitched crying. Authors^(15,35)^ discuss which mechanisms make breastfeeding can, in fact, relieve pain, with all this set of factors simultaneously responsible for relief. In addition to the comfort coming from the mother’s lap, skin-to-skin contact, direct suction on the breast, the distraction of the procedure and the sweet taste of breast milk, together, encompass this relief. Breast milk contains tryptophan, which is an amino acid precursor to melatonin; It is also associated with several metabolic functions, such as the synthesis of serotonin, which is responsible for feelings such as pleasure, reduction in anxiety levels, sleep regulation and improved mood^(15,35)^.

Breastfeeding is the most effective non-pharmacological pain relief intervention when compared to other methods alone, such as just holding the baby, making skin-to-skin contact, using topical anesthetics or cold sprays, music therapy, non-nutritive sucking, sweet-tasting interventions and expressed breast milk^(2)^. Therefore, in Theme 2, we observed that, according to the Belief Model^(19)^, nursing techniques, even with some knowledge about the benefit of breastfeeding during vaccination, have their actions limited by restrictive beliefs, which makes it difficult to practice and prevents pain relief to the newborn and infant.

In Topic 3 (Actions of nursing technicians), many of them reported not taking any action to relieve the pain of newborns and infants at the time of administering the vaccine because they believed that there was a lack of resources in the BHU. Few said they advise the mother to breastfeed at the time of administering the vaccine - this reinforces that babies continue to feel unnecessary pain, due to lack of guidance or encouragement from the health professional, characterized as a restrictive belief ^(2,7,12,36)^.

Recently, it was highlighted that health professionals (including nursing technicians) should encourage mothers to use breastfeeding as pain management during vaccine administration, as this is a basic human right^(19,32)^. According to the Technical Note from the Ministry of Health, the guidance is for those responsible for administering injectable vaccines to children to favor, support and encourage breastfeeding^(16)^.

The restrictive beliefs of nursing techniques impact their actions, preventing newborns and infants from receiving pain relief, even when the mother asks to breastfeed during this time. The Belief Model^(19)^ confirms that the health professional who does not allow breastfeeding for pain relief, despite being informed about the subject or having received a request from the family who wants to relieve the pain of their child, is negatively interfering with the practice. , with an overlay of his restrictive belief to the scientific evidence.

Regarding pharmacological guidance for pain relief, the prophylactic use of antipyretics before vaccines is no longer recommended, as it has the ability to inhibit the inflammatory response of the vaccine, interfering with the immune response and causing a reduction in the level of antibodies obtained through them. Because of this, the current recommendation is to use it with caution at the time of vaccination, and the risk-benefit must always be evaluated by the pediatrician^(36)^.

Some practices to reduce the pain and anxiety of the child and the family were recommended by the WHO^(26)^. As suggested, the health professional who performs the vaccination should avoid speeches that can promote anxiety or distrust in the newborn, in the infant, which is a dishonest attitude. There are actions that can be taken, including distraction and correct positioning for pain relief^(7,26,36)^.

Regarding the technique to apply the vaccine, the results of this research corroborate the practices and recommendations of the WHO^(26)^. Reception is one of the important ways to reduce the stress generated by the anticipation of pain and reduce anxiety, which can be more unpleasant than the pain itself, increasing its response^(7,36)^. In the present study, some nursing techniques demonstrated actions that facilitate reception, positively reinforcing the WHO recommendations.

### Study limitations

The study was carried out during the COVID-19 pandemic, a context that often-made scheduling data collection difficult and delayed. In addition, we consider the performance of data collection in only three BHUs limiting, as this could have been replicated in other health services and in other municipalities.

### Applications for clinical nursing practice

Considering that beliefs interfere in clinical practice, it is essential that nursing technicians receive formal training to align current practices in evidence-based primary care vaccine rooms, as recommended by the new Technical Note^(16)^. Such training is intended for these professionals to favor, support, and encourage the practice of breastfeeding during vaccine application as actions that will reflect on care, allowing more mothers to breastfeed their children during vaccination, so that their pain be significantly reduced.

## FINAL CONSIDERATIONS

The results of the study showed that the restrictive beliefs of nursing techniques in clinical practice have stood out from the scientific evidence, leading them to discourage or prevent the mother from breastfeeding during the application of the vaccine. It was also possible to conclude that, even after recent evidence proving that breastfeeding is the most effective method for pain relief during vaccination, newborns and infants continue to experience pain due to inadequate management practices.

We conclude that, despite the present knowledge and the facilitating actions presented by the nursing technicians, the understanding of the restrictive beliefs presented by them was essential to respond to the gap in the literature. Research sought to understand why health professionals - in this study, illustrated by nursing technicians -, despite being aware of the evidence on pain relief, continued to perform management inappropriately.

It is worth mentioning that the nurse’s role as an education agent for the nursing team is also essential to promote training and qualification of technicians and nursing assistants in the vaccine room, regarding the management of pain relief. With this, a better assistance to the newborn and infant during the moment of vaccination is sought.

This study, therefore, is original and unprecedented in research on neonatal and pediatric pain, in addition to being the first to endorse the new Technical Note of the Ministry of Health. As a final message, regardless of the belief that the health professional has, scientific evidence must prevail over belief, in a relevant way and put into clinical practice.
